# Case Report: Sacral Nerve Root Pelvic Neural Retraining, With Long-Term Sustainability After the Device Explantation

**DOI:** 10.3389/fresc.2021.655400

**Published:** 2021-07-27

**Authors:** Panteleimon Vassiliu, Filippos Patoulis, Leon Naar, Georgios Dendias, Nikolaos Arkadopoulos

**Affiliations:** ^1^4th Department of Surgery, Attikon University Hospital, National and Kapodistrian University of Athens, Athens, Greece; ^2^Massachusetts General Hospital, Harvard Medical School, Boston, MA, United States; ^3^Henry Dunant Hospital, Athens, Greece

**Keywords:** fecal incontinence, urinary incontinence, spina bifida, bladder detrusor muscle, neuroplasticity, neuronal remodeling, rehabilitation, SNM

## Abstract

**Introduction:** Sacral-Nerve-Neuromodulation (SNM) is an effective treatment increasingly used in patients with urinary (UI) and fecal incontinence (FI). The way it acts in the body at its full potential have not yet been elucidated. The authors review the literature on SNM and the way it possibly works, relating it to a case with an unusually favorable outcome.

**Case Presentation:** A female presented with UI and FI. Operated for meningocele as a neonate. It the age of 5 bilateral Cohen ureter reimplantation performed for persistent urinary infections due to vesicourethral reflux. At the age of 13, she started complaining about UI associated with a hyper functional detrusor muscle. After a diary incontinence evaluation with a standardized questionnaire, SNM was applied at the age of 18 and was retained for 4 years. She was re-evaluated with a yearly follow-up.

**Results:** The patient retained the positive effect of SNM even after its removal and in addition showed signs of improvement. The patient developed the sensation of fillingness of the bladder and the rectum, which she never had before the SNM implantation, sensation which she retained 29 months after (last follow-up) device removal, allowing her to control her voids.

**Discussion:** The modern literature hypothesis that SNM contributes to the plasticity of the nerves through the stimulated area is supported by the present case, in which the SNM effect remained and led to improvements even after its removal. Device settings are presented as they may correlate with the result. This reinforces and expands the frontiers of SNM application and research.

## Background

Neural tube defects, including bifid spine, occur in ~0.5–2/1,000 pregnancies worldwide (1). The plethora of patients with bifid spine suffer from urinary incontinence (UI) and/or fecal incontinence (FI). A common type of UI in these patients is the neurogenic bladder, which in 50% of untreated patients evolves to kidney damage and renal insufficiency by the age of 5 years (2). In a study of 518 participants with a bifid spine, 55.4% had FI, 76.3% had UI, and 46.9% had both types, with a profound negative effect on the quality of life of patients (3).

On one hand, UI and FI are treated, at least initially, with medications (anticholinergic), strengthening of the pelvic floor (Kegel exercises), various interventions (biofeedback), or surgery (bladder dilatation) (4). On the other hand, studies focusing on patients with double incontinence show that the treatment of choice is the neuromodulation of sacral nerve roots (SNM) with improvement rates ranging from 30 to 100% in medium-term follow-up (5, 6). This large percentage range is due to different criteria on patient selection and the predominant type of incontinence of selected patients. A survey of patients with urge UI associated with FI showed better results than in patients with predominant symptoms of FI (7, 8). On one hand, patients who do not improve significantly in the first year might experience a significant improvement later (9). On the other hand, there are few cases in the study in which patients, while improving in the first year, had a worsening of their symptoms after 5 years (9).

Although SNM is a treatment for UI and FI, it has not been elucidated completely, especially with regard to the aforementioned long-term effects. It has been suggested that SNM modulates the S3 and S4 sacral roots with involvement of the central nervous system and activation of spinal remodeling, which ameliorates the systole of the pelvic floor and rectal sensation (10–12). In order to add evidence to those mechanisms, we present a case that:

- Clearly demonstrates the effect of central neuroplasticity, involving sensation.
- Has a sustainable result verified at 29 months after SNM explantation. To our knowledge, this outcome at that duration has never been reported in the study, at least in the adult population.
- Has the specific, unusual tuning (impedance) of the SNM that may be, serendipitous, responsible for this result that is analyzed.

## Case

An 18-year-old female presented to our institution with congenital UI and FI. At neonatal age, she was diagnosed with sacrolumbar meningocele. On the fifth day after birth, she had a surgical repair of the meningocele. At that time, the urinary system of the patient was evaluated as anatomically intact by ultrasonography. She remained asymptomatic for 4 years. At the age of 4 years, she had intermittent urinary tract infections and was diagnosed with a high-pressure neurogenic bladder and bladder diverticula. She also had grade IV-V bilateral vesicoureteral reflux and renal pelvis dilation. Intermittent catheterizations were deemed necessary, as well as long-term antibiotic chemoprevention. At the age of 5 years, the condition was treated with bilateral Cohen's ureteral reimplantation (13) and bladder neck reconstruction, increasing the length of her urethra. She was under regular medical surveillance, while she continued to experience persistent urinary tract infections that were treated with anticholinergic medications, intermittent catheterization, and chemoprevention until the age of 12 years. At that time, she complained about UI and FI.

**Figure 1:**
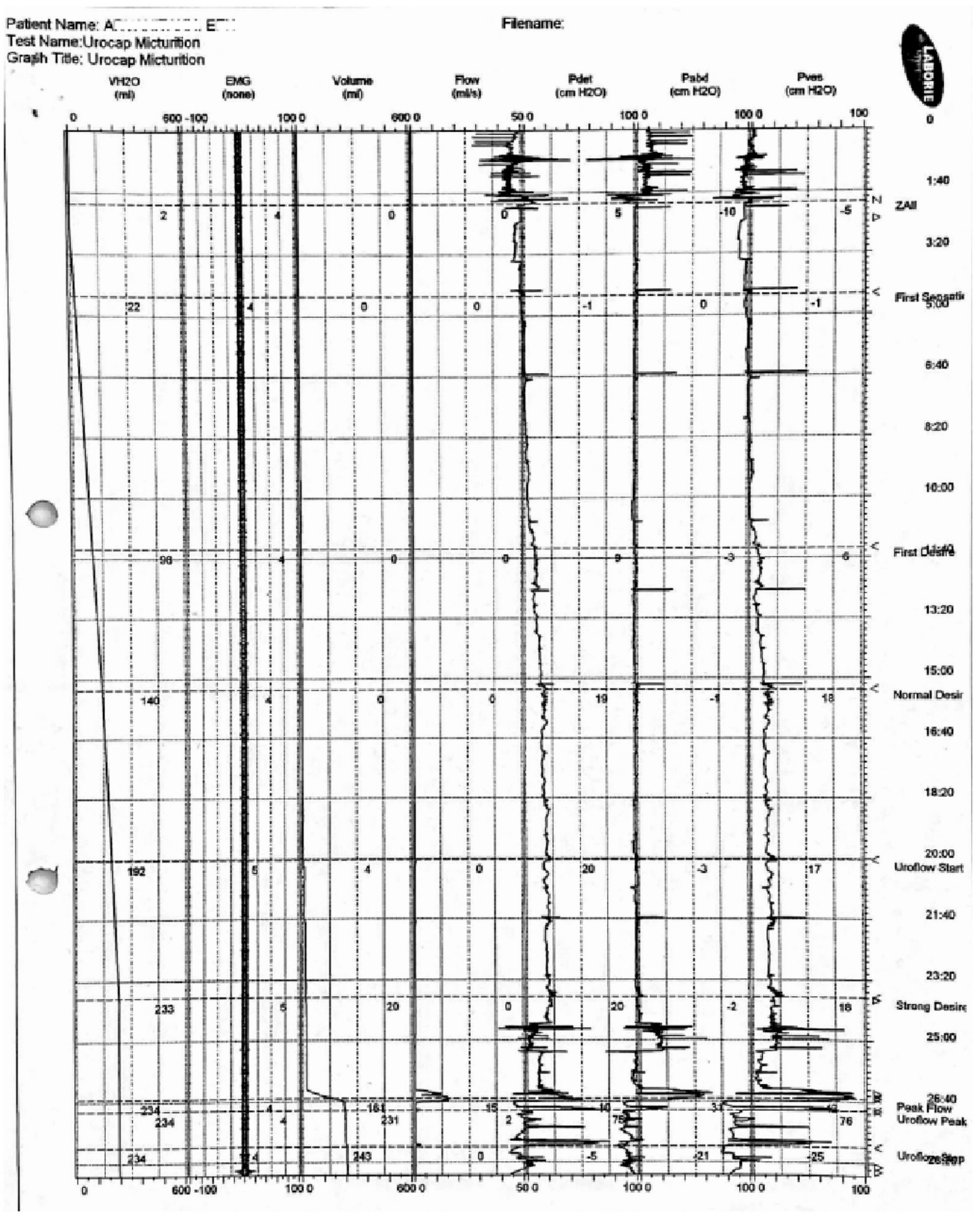
Related to the results reported in the text, the chart of the urodynamic analysis. This was the last evaluation before the SNM implantation (2010).

At the age of 16 years, she underwent ascending cystourethrography, where neurogenic bladder was identified with small pseudo-diverticula and a grade III right vesicoureteral reflux upon filling, which extended to grade IV during urination. In addition, several urodynamic analyses revealed diminished functional bladder capacity, reduced compliance of the bladder, overactive bladder detrusor muscle, involvement of abdominal walls in urination, and imperative incontinence: maximum flow = 15.3 ml/s, average flow = 6.5 ml/s, pressure at peak flow = 37.2 cm water (H_2_O), flow at peak pressure = 0.8 ml/s, peak pressure = 74.5 cm H_2_O, mean pressure = 21.8 cm H_2_O, P_det_Qmax = 15.7 cm H_2_O, P_det_max = 75 cm H_2_O, and P_ves_max = 91 cm H_2_O, P_abd_max = 66 cm H_2_O (Figure 1). The patient did not have a voiding cystourethrogram.

## Method

At the age of 18 years, the patient was referred to our department as an SNM candidate. Clinically, she had normal sensation and motor function after a complete neurologic examination. She had an atrophic sixth finger on her right hand and a mild right club foot. She completed a standardized 15-day voiding calendar for FI and UI. She changed diapers daily as many times as she urinated (48 diapers in 2 weeks, average 3.43 daily). Incontinence diary showed that 3.3% (1 in 30) of her defecations and 70.8% of her urinations were voided at the diaper, with a complete lack of rectal/bladder filling sensation and no control during sleep.

Afterward, the patient underwent implantation of a temporary neurostimulator. The most suitable foramen for the patient was evaluated between S3 and S4 foramens by artificial unipolar test stimulation lead (InterStim 3065 USC, Medtronic Inc., MN, USA), which was inserted at a 65° angle. Progressively augmented voltage was applied. The optimal location was decided based on sensation or in the absence of sensation of muscle contraction. The lowest possible voltage was the criterion of efficiency, allowing the selection of the optimal foramen. In this case, the S3 was chosen. The sensation was absent, and the patient instead had a clamp-like movement of the levator ani and plantar flexion of the great toe after an increase in voltage to 3 V. After the identification of the optimal foramen, the unipolar electrode was replaced by a temporary quadripolar tined lead (InterStim 3889, Medtronic Inc., MN, USA). The latter one was connected to the external test stimulator (InterStim 3625, Medtronic Inc., MN, USA). The temporary quadripolar tined lead was activated at the broad field (lead3: positive and lead0: negative). Stimulation frequency was set at 14 Hz and pulse width at 210 us. Ten days after the temporary implantation at her initial follow-up, as the anticipated sensation was not yet evident, the amplitude was increased at 4 V at which she developed numbness and a tingling sensation. The rest of the settings remained unchanged.

The follow-up was carried out at 6 months for the first year and yearly thereafter. It was comprised of a standardized 15-day calendar regarding her urinary and fecal voiding and all incidences of control loss, and evaluation of SNM settings in relation to patient sensory response, readoption of the stimulation settings according to patient response, and evaluation of the battery status.

## Results

During the test period, the calendar showed a 30% reduction in defecation frequency, while 4.8% (1 in 21 defecations) were incontinent. In addition, a 22.1% reduction in incontinence urinations was recorded. Although the margin of 50% improvement was not reached, based on the medical history of the patient and her willingness and perspective on SNM, it was decided to proceed to the permanent implantation, 27 days after initiation of the temporary neurostimulator. The temporary quadripolar tined lead was connected to the “InterStim 3058, Medtronic Inc., MN, USA” model stimulator.

On the first day after implantation, the stimulator settings were tuned at a frequency of 14 Hz with a pulse width of 240 us, and both remained constant throughout the stimulation period. The field was broadened among case/device: positive and lead0: negative. The amplitude was initially set at 0.8 V, as at this level the patient developed the desired tingling sensation. The stimulated field had an impedance of 627 Ω. The next day at the discharge of the patient, the voltage was dropped at 0.7 V and the field of stimulation was narrowed between lead2 (positive) and lead0 (negative). The change in the lead settings created a radical drop of impedance from 627 to 50 Ω and remained at that point through the whole duration of the therapy, which allowed 82 μA of current to run within the tissues. This impedance, although low, was above the limit of 25 Ω, which mandates a change in leads and was retained as the most comfortable adjustment for the patient. Those settings remained stable in the first year. During the next 3-year period, the SNM amplitude, based on the sensation of the patient, was set at 0.9 V (2nd and 3rd year) and 0.7 V (last year). The stimulated field remained constant between leads 2: positive and 0: negative.

Subsequently, the patient was constantly improving. In the 3-year follow-up after SNM implantation, no incontinence defecations were present and there was an improvement of 52.14% at the incontinence urinary voids compared to her condition prior to the test period. She also developed a sensation of bladder filling throughout the day and during sleep, the sensation that was non-existing at her initial assessment.

**Figure 2:**
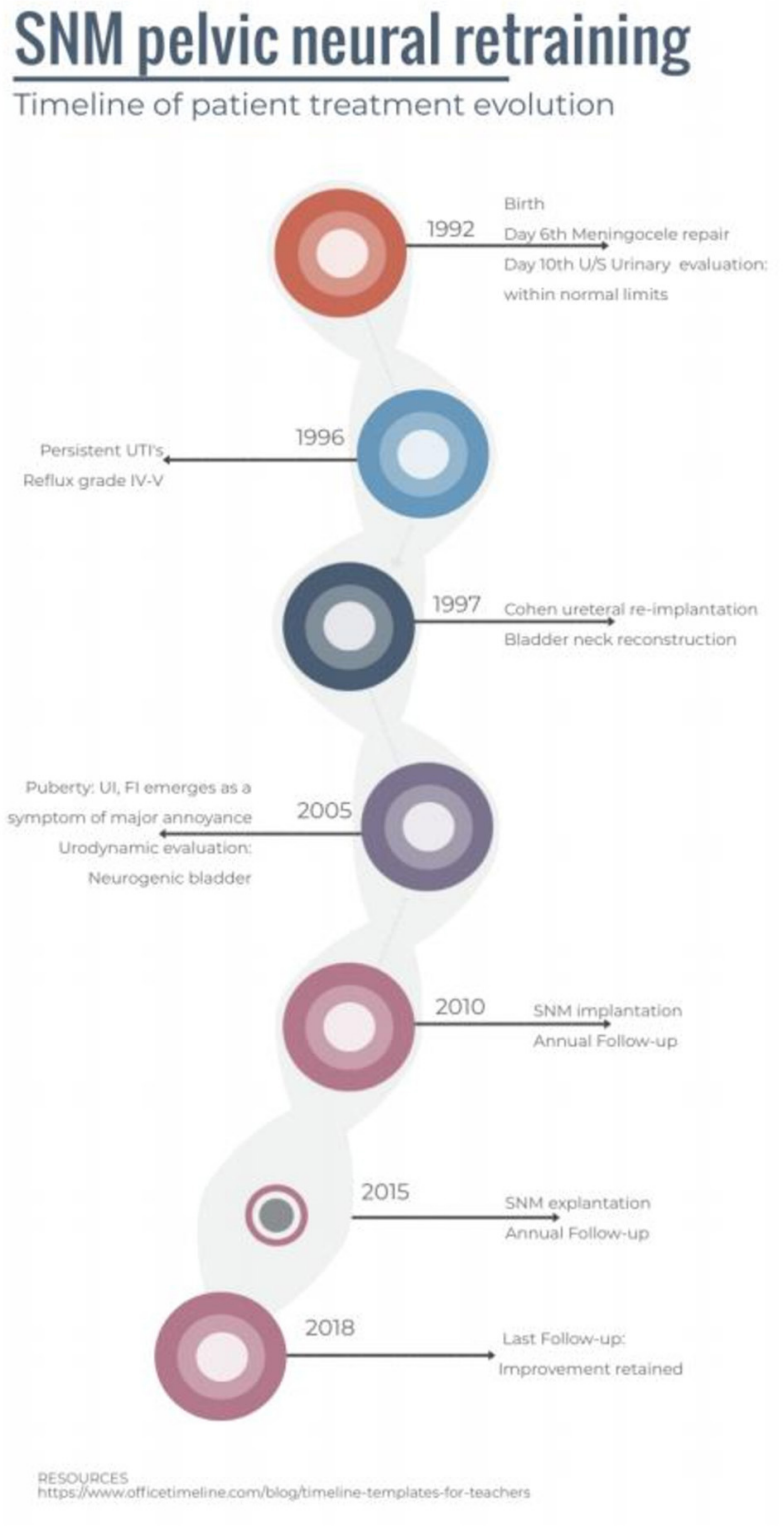
The complete timeline of the medical course of the patient.

Four years after the SNM implantation, as the patient was on a weight loss diet, the device had started eroding the overlying skin. In addition, the battery showed evidence of exhaustion, something that could be explained by the extremely low impedance that led to greater electrical current (82 μA) running through tissues. The stimulator was explanted, and a new one was ordered. The course of the patient is highlighted in Figure 2.

Until the approval of the new device order (delayed due to financial crisis in the country), more clinical evaluations were performed at 10, 17, and 29 months after explantation. In particular, in the last evaluation (29 months after explantation, October 2018) patient showed additional progress, as she had no FI (nor with watery feces), her UI had furthermore improved by 86.1%, and she used one diaper per day (Table 1). She had retained the 24-h bladder filling sensation.

**Table 1:** Evolution of patient's continent/incontinent urinations and number of diapers required daily.

	**Diapers per day (D)**	**Average number of incontinence urinary voids per day (I)**	**Average number of continence urinary voids per day (C)**	**Total daily urinary voidsT = I + C**	Percentage of daily incontinence urinary voids = I*100/T	**Improvement compared to the initial state of the patient in UI**
Initial stage of the patient	3.4	2.4	1.0	3.4	70.8%	Referral value^*^ corresponds to 100%
During PNE	4.2	2.3	1.9	4.2	55.2%	22.1%
Last tally with SNM on	1.4	1.4	2.7	4.2	33.9%	52.1%
17 months after turning SNM off	0.8	1.2	5.9	7.1	16.9%	76.0%
29 months after turning SNM off	1.0	0.7	6.5	7.2	9.7%	86.1%

*The patient has retained the 24-h filling sensation. Numbers in D, I, and C columns are calculated as a daily average from a 14-day calendar recording. The worth of notice is the increased number of controlled voids after the explantation of the SNM. Their frequency does not affect the quality of life of the patient. It may be attributed to the fact that, although the patient developed the filling sensation and she is aware of bladder and rectal content, she is not “trained” to defer the void function, which is acquired early in the life of a healthy individual*.

*UI, Urinary incontinence*.

* *referral value of UI (percentage of daily incontinence urinary voids) at the initial evaluation of the patient = 70.8%*.

## Discussion

**Figure 3:**
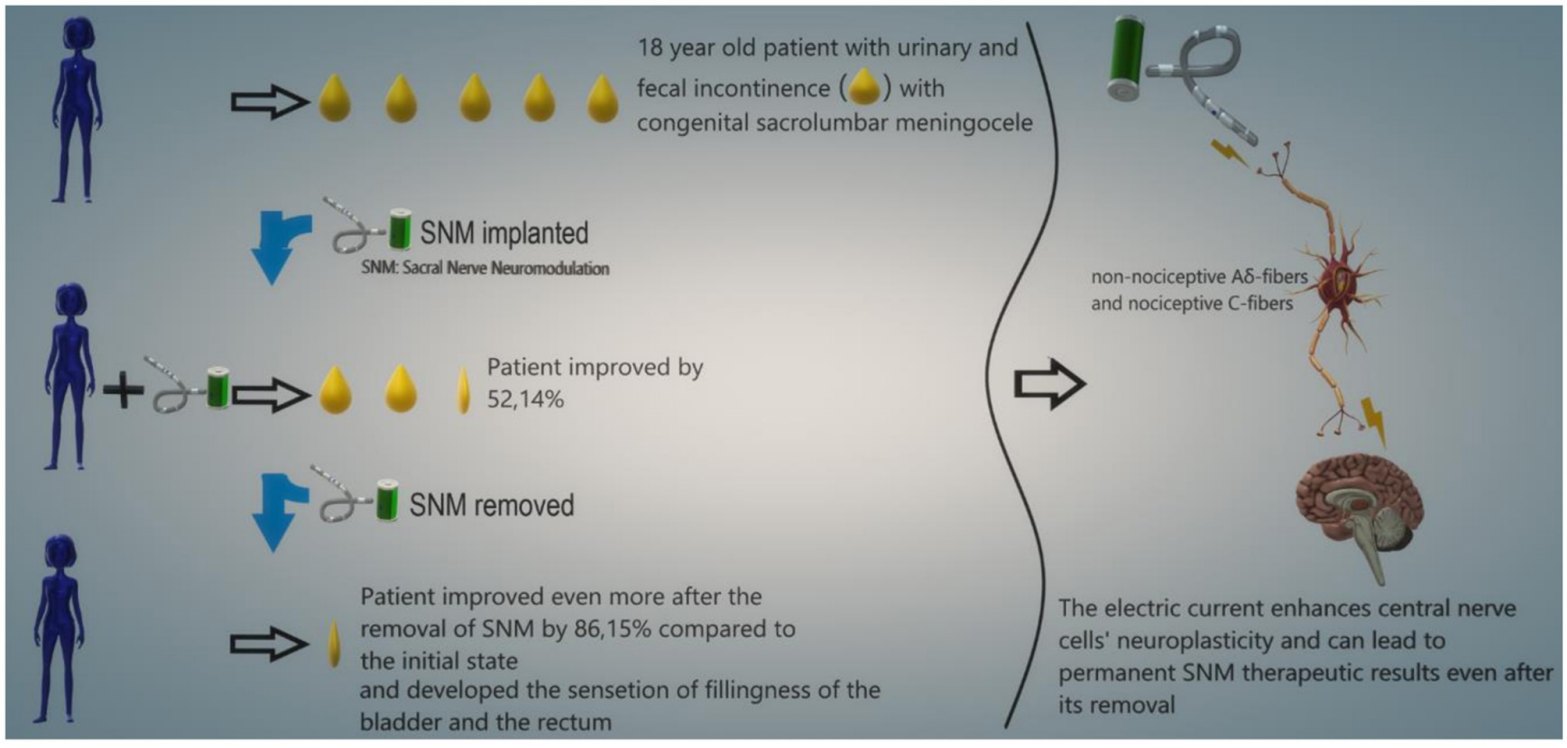
The timeline of the medical course of the patient since the implantation of SNM.

The use of electrical muscle stimulation prevents atrophy and stenghthen muscles, prompts neuroplasticity and obtains and maintains voluntary control (14). In addition, SNM causes central neuroplasticity through peripheral nerve stimulation (10–12). Evidence reinforcing this suggestion is offered from the evaluation of the real-time changes in brain activity during the application of SNM in patients with overactive bladder (15). The mechanisms *via* which SNM influences both bowel and bladder through the central nervous system are not well-known and even if they are comparable, it seems that they are not the same (10, 11). Investigating those functions in detail can enhance our understanding of how SNM works (Figure 3).

In contrast to conservative therapy, which usually targets only one kind of neurotransmitter receptor, the SNM seems to be targeting a variety of them (16). It is interesting that the positive effect of SNM against FI is not limited to direct changes in anorectal or sphincter function, as it was believed. On the contrary, as the modern study suggests, it covers a larger area of changes in neurotransmitters and synaptic functions (11). Studies have shown that in animals SNM affects bladder contractions through two different mechanisms: a peripheral effect during the stimulation and a central effect by neurotransmitters after the stimulation (11). The SNM tuning [amplitude (V), frequency (Hz), pulse width/burst time of electrical current (us), positioning of the electrodes, stimulated field (broad vs. narrow), and impedance (Ω)] can greatly affect the outcome of the results of this multi-transmitter (17).

The amplitude of SNM is set to a fraction of the engine limit (18, 19). A recent study has shown an “all or nothing” phenomenon when the SNM voltage is set above the half stimulating threshold of the motor cortex center of the brain of the anus showing significant improvement (*p* < 0.001) relative to intensities below half of this threshold. Further increases in intensity did not result in significant improvement (18). In clinical practice, we evaluate the brain indirectly through sensory or motor responses of the perianal area. Our awake patient during the implantation of the temporary neurostimulator under local anesthesia had a remarkable absence of the perianal tingling sensation, so we were obliged to use the criterion that applies to anesthetized patients, that is the perineal/anal muscle contraction. Thus, we reached 3 V to have muscle contraction, which the patient tolerated with comfort.

The use of SNM at pulse widths of 180 and 240 us and a frequency of 14 Hz is suggested (18). However, these values differ in other mammals (19). In our practice, we use 210 us in the evaluation period and 240 us ever after. It is supported in the study that different excitation frequencies release different neurotransmitters in the first vertebral synapses between the primary axonal and neuronal primers (20). In this case, the frequency was maintained at 14 Hz, throughout the stimulation period.

Regarding electrode positioning, incorporating four electrodes as close as possible to the lumbar S3 nerve has been suggested, (21) as ~70% of the external pressure of the urethral sphincter is dependent on the excitatory activity of the S3 root, while S2 and possibly S4 contribute to the remaining 30% (22). Incorrect placement of the electrodes can have a negative effect on the patient, due to stimulation of nerves irrelevant to the ones participating in incontinence (21, 23). In our case, we used a four-lead tip electrode implanted at S3.

The functional MRI revealed that larger stimulus intensity (3.7 ± 1.1 V) influences greater and various regions of the brain and that the changing stimulus intensity modifies the pattern of cerebral activity (15). A recent study shows that any size of intensity can cause additional electromagnetic waves by afferent neural pathways, which activate spinal motoneurons (14). In addition, a research study indicates that the interaction between alterations of intensity and the afferent pathways determines the activation of spinal motoneurons to the corresponding plane (14). Our patient was not exposed to an increased amplitude (0.7–0.9V), and this may indicate that amplitude may not be the only cause of this extraordinarily good result.

The impedance initially was 627 Ω. However, after the field of stimulation was narrowed between lead2 (positive) and lead0 (negative), the impedance was greatly diminished and maintained <50 Ω throughout the stimulation period. This low impedance is not considered a short circuit, and according to our programmer device measurements allowed 82 μA of current to run between the leads within the tissues. Every other combination of positive and negative leads had impedance >361 Ω and <15 μA. Given the excellent clinical course of the patient under this very low impedance, we can hypothesize that we created a serendipitous tight connection with neural tissue around S3 through which high electrical current was transmitted, neuromodulating the central nervous system. Therefore, impedance may play an uninvestigated role in SNM. This low impedance results in earlier battery exhaustion due to the high current flow, but the clinical course of the patient overcomes this consequence.

**Figure 4:**
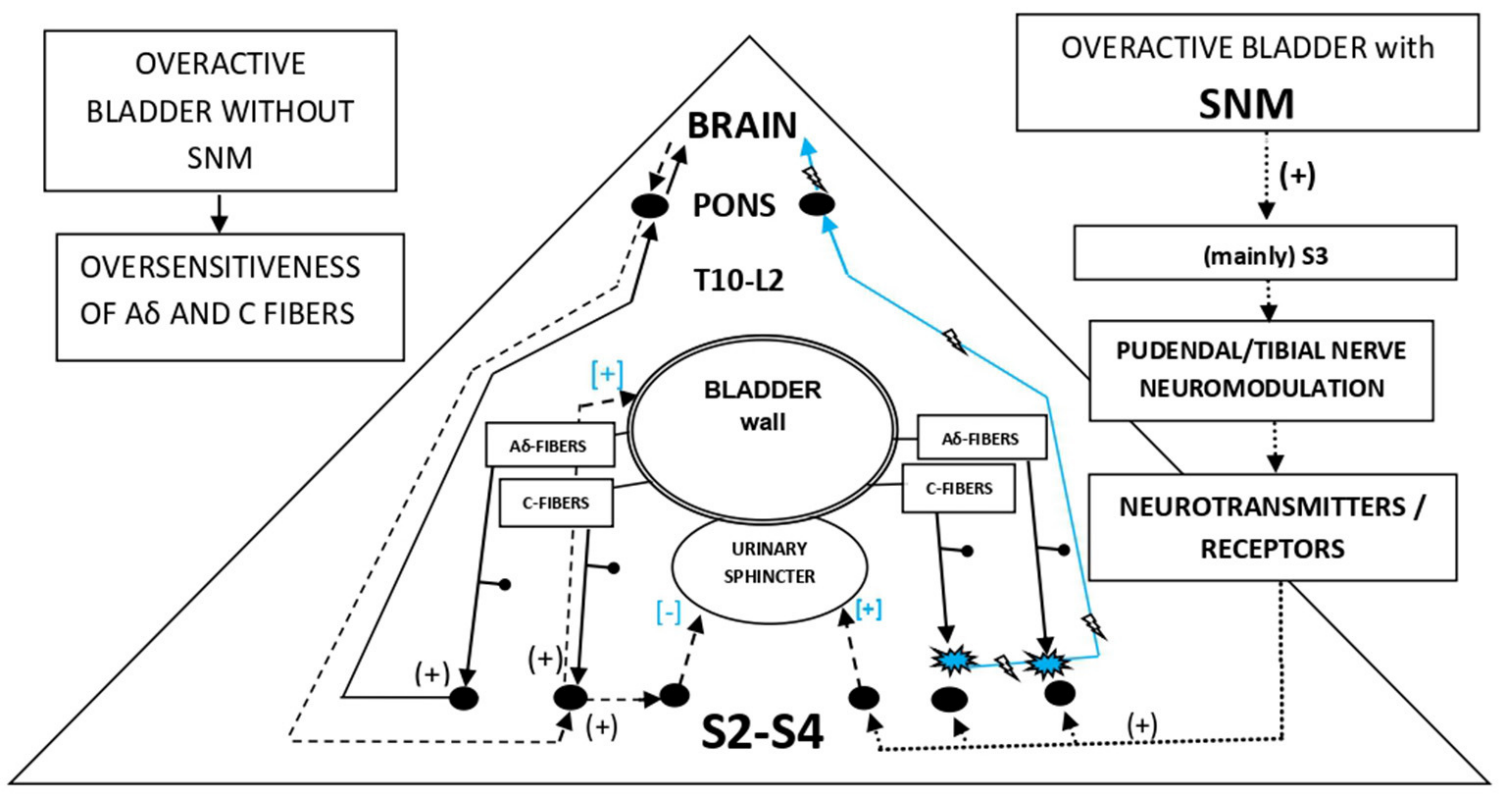
Possible mechanism of SNM effect on urinary control.

The current case reinforces the observation that the SNM contributes to the plasticity of the nerves of the stimulating area, a plasticity retained even after the deactivation of SNM. Not only did the patient clinically improve and continue her good clinical course with a gradual reduction in her incontinence but also at the same time she developed characteristics (bladder filling sensation) that were absent before SNM (Figure 4). It remains to be proven whether SNM tuning (low impedance and high μA) or manipulation of other parameters can maximize this neuroplasticity effect.

Regarding the retaining of the positive effect of the SNM even after its discontinuation, a study followed 19 people with FI and/or UI who had SNM for at least 1 year. SNM of those patients was turned off. In nine patients, the advantages of SNM were maintained. In the rest 10, some symptoms recurred after different periods and their SNMs were reactivated. No correlation of incontinence relapse was found with the evaluated risk factors. However, the severity of the initial disease, its idiopathic etiology, and UI had increased chances for relapse. The chances of retaining the benefits of SNM diminish with time (from 95 to 74% after the first month and from 67 to 55% after 6 months). The sixth month is indicated as the critical month of sustainability of the positive effect of SNM (12). In comparison to these elements, according to our knowledge, our patient has been followed up for 29 months without any sign of relapse, a duration noticeably longer than any other reported in the study, at least in the adult population. In the study of Altomare et al., only 3 out of 19 patients retained the benefit of SNM 1 year after the explantation of the device and there was no follow-up after that (12). Identifying which SNM parameter repeatedly creates this long-term effect would have a remarkable impact on the outcome of these patients.

Our study reinforces the expectations raised by Rensing et al., who demonstrated the possibility of discontinuing SNM under certain conditions in pediatric patients (median age at implantation of SNM was 10.1 years), as a possible strategy for the cure. In particular, in children with at least 2 years of SNM use and good treatment response, they showed that the chance of long-term successful removal of SNM is directly proportional to its duration of use (24). Our case shows this possibility also in the adult population as well. However, clinicians should always bear in mind that this has to be demonstrated in a larger number of patients and that there is always the possibility of relapse.

Limitations of this case report include the lack of electromyography at the initial evaluation since her neurologic clinical examination was within normal limits. At follow-ups, a urodynamic examination or a voiding cystourethrogram could be added, which would evaluate the condition of the detrusor muscle spasticity, compliance, sensory threshold for urination, and urine residue after voiding. This was proposed at the last follow-up. If it were carried out, it would offer proof of the potential neuroplasticity results on bladder motor function, and although this was explained to the patient, she refused to have it.

## Patient Perspective

The patient stated exhilaratingly that “When I used the device for the first time, I did not see much improvement. I hoped that my doctors would be right, and my bad condition would change soon. Indeed, they were. After 3 years, I decreased the number of diapers from four to two, and even to one per day. When they told me that the device might not work because of the lack of the battery, I feared my bad situation may recur. Surprisingly, it did not. Contrariwise, it was improved, and my incontinence continued to be diminished. My doctors were surprised too! I was the first case they had ever known with such an amazing outcome! Today, I continue improving. I have developed the sensation of fillingness of the bladder and the rectum, which I never had before. It changed my life.”

## Conclusion

Currently, except for the established principle of nerve stimulation that enhances a motor response, additional benefits result in neuromodulation. With the current case, we add to the body of evidence that the electric current running through neural tissue may possibly enhance neuroplasticity of central nerve cells and can lead to permanent SNM therapeutic results even after its deactivation.

## Data Availability Statement

The original contributions presented in the study are included in the article/supplementary material, further inquiries can be directed to the corresponding author/s.

## Ethics Statement

Ethical review and approval was not required for the study on human participants in accordance with the local legislation and institutional requirements. The patients/participants provided their written informed consent to participate in this study. Written informed consent was obtained from the individual(s) for the publication of any potentially identifiable images or data included in this article.

## Author Contributions

PV: conception, design, interpretation, editing the article, and treating physician. FP: acquisition, data analysis, and drafting the text. LN: review and draft article. GD: review article. NA: review and editing the article. All authors contributed to the article and approved the submitted version.

## Conflict of Interest

The authors declare that the research was conducted in the absence of any commercial or financial relationships that could be construed as a potential conflict of interest.

## Publisher's Note

All claims expressed in this article are solely those of the authors and do not necessarily represent those of their affiliated organizations, or those of the publisher, the editors and the reviewers. Any product that may be evaluated in this article, or claim that may be made by its manufacturer, is not guaranteed or endorsed by the publisher.
